# The prevalence of suicidal ideation and depression among primary care patients and current management in South Korea

**DOI:** 10.1186/s13033-017-0123-9

**Published:** 2017-02-07

**Authors:** Yoon-Joo Choi, Weon-Young Lee

**Affiliations:** 1 Department of Preventive Medicine, College of Medicine, Chung-Ang University, 84 Heukseok-ro, Dongjak-gu, Seoul 156-756 South Korea; 2 Department of Preventive Medicine, College of Medicine, Chung-Ang University, 84 Heukseok-ro, Dongjak-gu, Seoul 156-756 South Korea

**Keywords:** Primary care, Prevalence, Suicide, Depression, Self-reported, Questionnaire

## Abstract

**Background:**

Primary care in South Korea has no effective screening system to identify high-risk suicide patients despite to the possibility of hidden patients. The present study examined the prevalence of suicidal ideation and depression among primary care patients and investigated rates of recognition and management strategies of physicians as they encountering patients with suicidal ideation and depression in primary care settings.

**Methods:**

This study was conducted as a two-part survey of patients visiting primary care clinics and their physicians. (1) The survey for patients was administered over 17 days in two areas and assessed socio-demographic characteristics, health behavior and the prevalence of suicidal ideation and depression. The participants were 1363 outpatients; 848 lived in urban area, and 515 were from rural area. (2) We surveyed the physicians’ recognition of patients with suicidal ideations and depression as well as their current management. Eighteen doctors at 15 local clinics (8 in urban area and 7 in rural area) participated in this survey.

**Results:**

The prevalence rates of suicidal ideation and depression (Patient Health Questionnaire-9 ≥ 10) were 18.3% (95% confidence interval: 16.2–20.3) and 13.9% (95% CI 12.6–15.7), respectively in primary care settings. The rates of suicidal ideation and depression were approximately 2.4 times and 1.4 times higher, respectively than those in community dwelling people. Ten (69.7%) and 4 (26.7%) of the 15 clinics staffed physicians who did not recognize suicidal ideation and depression, respectively. Five (83.3%) of 6 and 4 (38.6%) of 14 physicians who recognized suicidal ideation and depression among their patients respectively, only recommended psychiatry without any arrangements for a referral.

**Conclusion:**

Our findings imply that many patients with suicidal ideations and depression in primary care settings are under-diagnosed and under-treated. As a result, education and training of the identification and management of suicidal ideation and depression should be made available to physicians in primary care settings.

## Background

South Korean suicide rates have been the highest among Organization for Economic Co-operation and Development (OECD) member states since 2004 [1]. Although the Korean government has made great efforts to reduce these rates, suicide still remains serious public concern among Korean people. A previous systematic review study of suicide prevention strategies stressed the role of primary health care in effective suicide prevention [2]. Given that primary care is typically the first contact point for most patients into the health care system and is more accessible than psychiatric clinics or psychiatric hospitals. Primary care physicians are the most likely providers to see patients with mental health problems, including suicidal ideation or depression [3–5]. Moreover, many suicide victims had documented contact with a general practitioner in a primary care setting in the weeks before their act of suicide [6, 7]; therefore, it could be ideal that patients with suicide ideation should be screened and start some type of treatment in the primary care setting [8]. In particular, suicide prevention in primary care should focus on the recognition and treatment of depression because it is a major risk factor of suicide and one of the most common mental disorders encountered in primary care [3].

Primary care in South Korea has only a minor role to play in suicide prevention [9], because no suicide prevention education programs currently exist for physicians in primary care settings at government level. Some studies have shown that similar education programs can lower suicide rates and increase the detection of suicidal ideation and depression as well as effectively manage psychological problems in primary care [10–13]. Second, primary care physicians in Korea are allowed to provide specialist care as well as generalist care as the first point of call and also may refer patients to specialist; they also have private ownership of their health care facilities. Moreover, many physicians in primary care settings are specialists, including internists, pediatricians, family doctors, OB/GYN, general surgeons and psychiatrists.

Under fee-for-service reimbursement system of Korean National Health Insurance (KNHI), most primary care physicians are therefore very busy providing more consults and diagnostic service with low technology to patients with physical health problems to generate more money [14].

In this context, the management of suicide ideation and depression in primary care would not be an object of concerns for many practitioners at local clinics except for psychiatrist. Unfortunately, due to stigmas surrounding psychological disorders, many patients are not willing to visit a psychiatrist [15]. Considering the high suicide rates of Korea among all OECD member states, it is likely that patients with suicidal ideation or depression are prevalent in local clinics. However, to the best of our knowledge, little research has been conducted to determine the prevalence of suicidal ideation and depression among primary care patients in Korea. Therefore, this study aimed to examine the prevalence of suicidal ideation and depression among primary care patients compared with general population. Furthermore, we investigated whether physicians at local clinics could recognize that their patients suffered from suicidal ideation or depression and if they knew how to act when they encounter such patients.

## Methods

### Sample

The Gu region in Seoul and the Si (cities) area in Kyonggi-do were selected for this study. The former is a metropolitan area of approximately 0.32 million population, while the latter is a mix of urban and rural areas in the suburbs of Seoul with 0.1 million people. The Public Health Center (PHC) in each area, which is responsible for local health administration and provides outpatient medical services for mild illness, recruited local private clinics that were willing to to take part in our survey. In the Gu area, eight sites, including the PHC, were recruited; the physicians who worked there were specialized as follows: general medicine (4), which indicated a medical degree but no speciality–, internal medicine (4) and Korean traditional medicine (2). Six of the eight facilities that took part in this survey were solo practices, and the other two sites employed two physicians each. Seven sites, including the PHC in the Si area were selected for our survey, and the specialties of all physicians at those clinics were as follows: general medicine (2), internal medicine (2), rehabilitation medicine (2), and Korean traditional medicine (2). Six of the seven facilities were solo practices, and the remaining site had two physicians.

**Fig. 1:**
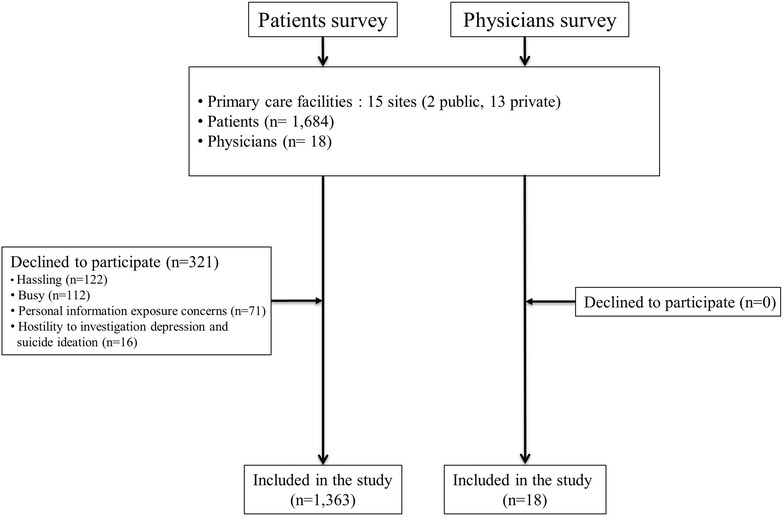
Flow chart of patients’ and physicians’ survey in primary care settings

The number of daily visits by patients per site varied between 20 and 80. A trained interviewer attended a certain clinic between 10 a.m. and 5 p.m. on 1–2 working days each week depending on the number of daily patient visits. The survey was conducted for 9 working days in the Si on October, 2015 and for 8 days in the Gu on November in the same year. On these days, all patients were asked in consecutive order whether they were willing to take part in our survey; 1363 (83.4%, Gu: 75.9%, Si: 90.8%) of the 1635 eligible outpatients aged 20 and over who sought treatment for physical illness or a health check-up agreed to participate (Fig. 1). The reasons why 272 outpatients declined to participate were: a hassle (38%), too busy (35%), concerns about being personal information exposed to the public (22%), and a hostile attitude to investigations of mental health (5%). The questionnaire for the participants assessed their socio-demographic characteristics, health behaviors and mental health using the Patient Health Questionnaire-9 (PHQ). To compare this study with the prevalence rates of suicidal ideation and depression in the general population, we analyzed data from the 2013–2014 Korean National Health and Nutrition Examination Survey (KNHANES) which used the PHQ to assess the mental health status of people in the community.

During the same period of the patient survey, 16 physicians who interacted with the patients of all the clinics involved in this study were asked about their experiences of meeting patients with suicidal ideation or depression and how they treated such cases at that time. All physicians took part in our survey.

### Instruments and the PHQ-9

The patient questionnaire consisted of three parts. The mental health section contained the PHQ-9 which is a multipurpose instrument for screening, diagnosing, monitoring and measuring the severity of depression and suicidal ideation [16, 17]. It is brief and useful in clinical practice and incorporates the Diagnostic and Statistical Manual (DSM-IV)’s diagnostic criteria for depression with other leading major depressive symptoms into a brief self-report tool. The depression module scores for each of the nine DSM-IV criteria from “0" (not at all) to “3" (nearly every day). PHQ-9 scores of 5, 10, 15, and 20 represented mild, moderate, moderately severe, and severe depression, respectively.

Question 9 on the PHQ-9, “Having thoughts that you would be better off dead, or of hurting yourself on at least 2 days over the past 2 weeks,” screens for the presence and duration of suicidal ideation if rated as 1 point or more. The Korean version of the PHQ-9 has an acceptable reliability and validity [18]. The demographic section gathered information regarding participants’ age, gender, religion, marital status, education, and occupation. A final section about health behavior consisted of subjective health status, reasons for visiting primary care, past history of mental illness and current treatment of mental illness.

### Statistical analysis

We calculated the prevalence rates and their confidence intervals for patients with suicidal ideation or depression in primary care by severity and age group in both sexes using our survey data and also the KNHANES data. Next, differences between patients both with and without suicidal ideation were examined for their demographic variables and health behavioral factors using the univariate student *t* test and χ^2^ tests. Moreover, student t tests were run to determine differences in the prevalence rates of patients with suicidal ideation or depression in primary care between local clinics where physicians identified mental health status in their patients and local clinics where physicians did not know it at all. Finally, we determined the frequency of the methods patients used to treat patients with suicidal ideation or depression as they encountered them in clinical practice. The data analyses were conducted using STATA 13.0 using statistical significance being set at P ≤ 0.05.

## Results

The 2 week-point prevalence rates of suicidal ideation and depression (PHQ ≥ 5) among patients visiting local clinics were 18.3% (95% CI 16.2–20.3) and 36.6% (95% CI 34.0–39.2), respectively, while those in the general population were 7.7% (95% CI 6.9–8.4) and 22.1% (95% CI 21.0–23.2), respectively (Table 1). Of the 1363 primary care patients, 12.0% (95% CI 10.3–13.7) were bothered by suicidal ideation on some days, 3.9% (95% CI 2.9–4.9) on more than half of the time and 2.4% (95% CI 1.5% to 3.1) on almost all days of the last 2 weeks (Table 1). The prevalence rates of suicidal ideation in primary care patients who reported a daily frequency of suicidal thoughts were around 1.4–3.3 times higher than those in the general population in all groups. The prevalence rates of depression by severity based on the PHQ in primary care settings were as follows: mild (25.1%, 95% CI 22.8–27.4), moderate (7.0%, 95% CI 5.6–8.3), moderate severe (2.7%, 95% CI 1.8–3.5), and severe (1.8%, 95% CI 1.1 to 2.5), as shown in Table 1. The prevalence rates of depression by disease severity in primary care were 1.4 times higher than those of depression by disease severity in the general population (Table 1). After a comparison by age group, the suicidal ideation prevalence in primary care were found to be around 1.4–3.3 times higher than that in the community across all age groups. In addition, the prevalence rates of depression in primary care by severity were approximately 1.4–1.8 times higher than those in the community (Table 1).

**Table 1:** Prevalence of suicidal ideation and depression using PHQ-9 in primary care and general population

Survey	Survey for participating local clinics (N = 1363)						2013–2014 Korean national health and examination survey (community people) (N = 6037)					
Tool	PHQ-9 Korean version						PHQ-9 Korean version					
Classification	Suicide ideation			Depression			Suicide ideation			Depression		
	Male	Female	Total	Male	Female	Total (95% CI)	Male	Female	Total	Male	Female	Total (95% CI)
Severity^a^												
Severe	1.6	2.8	2.4 (1.5, 3.1)	1.0	2.2	1.8 (1.1, 2.5)	0.1	1.0	1.5 (1.2, 1.8)	0.6	2.3	0.7 (0.5, 0.9)
Moderate severe	3.3	4.2	3.9 (2.9, 4.9)	1.6	3.3	2.7 (1.8, 3.5)	0.7	2.3	1.0 (0.7, 1.3)	0.4	1.5	1.6 (1.3, 2.1)
Moderate	12.0	12.0	12.0 (10.3, 13.7)	4.7	8.4	7.0 (5.6, 8.3)	3.1	5.6	5.2 (4.6, 5.8)	4.3	6.2	4.5 (4.0, 5.2)
Mild				23.5	26.0	25.1 (22.8, 27.4)				11.8	17.9	15.3 (14.3, 16.3)
Cumulative sum			18.3 (16.2, 20.3)			36.6 (34.0, 39.2)			7.7 (6.9, 8.4)			22.1 (21.0, 23.2)
Age group												
18–29	15.0	15.1	15.1 (13.2, 17.0)	32.5	45.3	39.8 (37.2, 42.4)	7.3	7.8	7.6 (6.9, 8.3)	26.8	34.7	29.4 (28.1, 30.7)
30–39	10.0	11.8	11.3 (9.6, 13.0)	35.0	33.3	33.8 (31.3, 36.3)	3.9	4.2	3.9 (3.4, 4.4)	21.5	27.6	23.9 (22.7, 25.1)
40–49	21.4	10.7	14.3 (12.4, 16.2)	28.6	30.9	30.2 (27.7, 32.6)	3.2	5.1	4.3 (3.7, 4.9)	18.5	20.2	17.9 (16.8, 18.9)
50–59	11.0	14.5	13.2 (11.4, 15.0)	21.0	32.0	27.9 (25.5, 30.3)	3.9	9.6	7.2 (6.5, 7.9)	14.6	24.0	19.2 (18.1, 20.3)
60–69	6.8	16.3	13.1 (11.3, 14.9)	19.4	37.9	31.7 (29.2, 34.2)	5.6	12.8	9.3 (8.5, 10.1)	12.2	25.3	20.3 (19.2, 21.4)
70+	25.9	27.4	26.9 (24.5, 29.2)	42.2	48.1	45.9 (43.2, 48.5)	8.3	20.2	14.6 (13.6, 15.6)	14.0	32.2	25.8 (24.6, 27.0)
Total average	18.3	18.3	18.3 (16.2, 20.3)	42.2	36.6	36.6 (34.0, 39.2)	5.4	9.9	7.7 (6.9, 8.4)	17.0	22.3	22.1 (21.0, 23.2)

^a^Suicide ideation assessed by question #9 of PHQ-9 and depression assessed by Total Scoring./Suicide ideation (Mild) several day (Moderate) More than half the days (Severe) Nearly every day/Depression (Mild) Total scoring 5–9 (Moderate) 10–14 (Moderate severe) 15–19 (Severe) 20–27

The differences in the characteristics between patients with and patients without suicidal ideation were presented in Table 2. Univariate testing of these differences revealed that patients with suicidal ideations were significantly more likely to be in female, unmarried, to have little education and to live alone than were patients without suicidal ideations. In addition, patients in poor subjective health who had a history of a mental disorder and complained of depressive symptoms (PHQ ≥10) significantly suffered from suicidal ideation more often than would otherwise be the case. Patients with suicidal ideation were also significantly more likely to see a doctor for acute and chronic physical illness than patients without suicidal ideations.

**Table 2:** Univariate analysis of the differences between patients with and without suicidal ideation (N = 1363)

	No suicidal ideation (n = 1114)	Suicidal ideation (n = 249)	χ^2^	p
Demography				
Mean age in years (S.D.)	61.6 (15.2)	68.4 (14.5)	−6.476^a^	≤.001
Female/male	63.5%/36.5%	66.7%/33.3%	0.91	0.341
Married/unmarried or divorced, bereaved	65.5%/34.5%	43.0%/57%	78.73	≤.001
Lower education/higher education	52.7%/47.3%	72.2%/27.8%	68.54	≤.001
Urban area/rural area	62.8%/37.2%	59.4%/40.6%	1.00	0.317
Living alone/living together	20.0%/80.0%	36.3%/63.7%	49.75	≤.001
Health status				
Good/bad subjective health status	73.9%/26.1%	36.1%/63.9%	155.67	≤.001
Never/had diagnosed mental illness	91.9%/8.1%	78.1%/21.9%	44.34	≤.001
Depression (PHQ-9 ≥ 15)/not depression	0.5%/99.5%	22.1%/77.9%	587.41	≤.001
Reason for seeing a doctor				
Physical illness (acute)	79.1%	20.9%	457.61	≤.001
Physical illness (chronic)	75.3%	24.7%		
Medical check up	88.9%	11.0%		
Others	95.7%	4.3%		

^a^T value by student *t* test

As shown in Table 3, of the 15 local clinics involved in this study, physicians at 5 clinics (30.3%) recognized that their patients were suffering from suicidal ideations and physicians at 11 clinics (73.3%) recognized that their patients had depression. Differences in the average prevalence rates of depression and suicidal ideation between clinics with physician recognition and clinics without physician recognition were not significant according to the student *t* test. The average prevalence rates of suicidal ideation and depression in the 10 and 4 clinics, respectively, where their doctors did not recognize these conditions, were 16.1% (±3.0) and 25.5% (±10.4). Of the six physicians who encountered patients with suicidal ideations in clinical practice, five (83.3%) recommended a psychiatry visit near the patients’ residence and one (16.7%) tried to treat them and only recommending a psychiatry referral if the condition worsened. Ten (71.4%) of the 14 physicians who identified patients with depression in clinical practice, tried to treat the condition themselves and advised patients to attend a psychiatrist only if they did not improve. The remaining four (38.6%) physicians recommended visiting a psychiatry specialist near the patients’ residence.

**Table 3:** Difference in the average prevalence rates of suicidal ideation and depression in local clinics according to the physician’s recognition of them and the physician’s actions whens encountering such patient

Physicain’s recognition of suicidal ideation and depression among his or her patients	Number of local clinics	Average prevalence rates in local clinics (S.D.)	t-value (p value)
Suicidal ideation			
Yes	5	19.5 (4.0)	1.662 (0.122)
No	10	16.1 (3.0)	
Depression (PHQ ≥ 10)			
Yes	11	13.7 (7.3)	1.702 (0.112)
No	4	7.2 (3.2)	

Physician’s act as encountering patient with suicidal ideation and depression if physician recognize it	Percentage (number of doctors)
Suicide ideation (N = 6)	
Recommendation of visiting a psychiatrist without any arrangement	83.3% (5)
Treatment and recommendation of visiting a psychiatry if it worse	16.7% (1)
Depression (N = 14)	
Recommended to visit a psychiatrist without any arrangement	28.6% (4)
Treatment and recommended to visit psychiatry if it worse	71.4% (10)

## Discussion

The prevalence rates of suicidal ideation and depression (PHQ ≥ 10) in primary care settings were approximately 2.4 times higher (18.3%, 95% CI 16.2–20.3) and 1.4 times higher (36.6%, 95% CI 34.0–39.2) than those in the general population, respectively. These patterns were similar in all severity and age groups. From clinical practice viewpoint, suicidal ideation was observed significantly more often in patient with poor subjective health, a history of psychological illness, a depressed status, and in those who sought help for physical illness than in all other participants. Even though a considerable number of primary care patients suffered from suicidal ideation or depression, 10 (69.7%) and 4 (26.7%), respectively, of the 15 total clinics employed physicians did not recognize these conditions in their patients. Five (83.3%5) of the 6 physicians who identified suicidal ideation among primary care visitors and 4 (38.6%) of the 14 physicians who recognized depression among primary care visitors recommended a psychiatry consultation without any arrangement.

The existent literatures have reported that the prevalence rates of suicidal ideation and depression are much higher in primary care settings than in the general population [19, 20]. This study produced similar results. Compared to a general population, it can be assumed that primary care visitors are more likely to have poor mental health, because of their health status. These results imply the possibility of primary care’s key role in effective suicide prevention.

18.3% of primary care visitors in our sample responded affirmatively to the suicidal ideation item of the PHQ. This figure is higher than the 12% reported by a German primary care study and the 7–8% presented by two United States primary care studies that used PHQ to assess suicidal ideation [19, 21–23]. Several explanations might account for this finding. Patients who were excluded from our data because they declined to participate in our survey might be less likely to present poor mental health. However, considering that the cited reasons for declining to participate included concerns about personal information being exposure to the public or hostility to any type of psychiatric investigation, there may have been selection bias that led to an overestimation of the prevalence of suicidal ideation. There would be another explanation finding for our findings. Given that Korea has ranked the first place among OECD member states for 12 years in suicide rates, the prevalence rates of suicidal ideation in Korea might be relatively much higher than any other country.

According to Kroenke et al’s study [16], scores less than 10 seldom occur in individuals with major depression whereas scores of 15 or greater usually signify the presence of major depression, with the gray zone including scores from 10 to 14. Considering that the prevalence of scores 15 or higher and 10 or greater were 4.5 and 13.9%, respectively, in this study, it is presumed that the prevalence of major depression in primary care is likely far higher than the 4.5% based on a provisional diagnosis using PHQ-9 scores. This prediction is supported by a previous Korean study [24], which used a diagnostic interview that was conducted by psychiatrists following the DSM-IV criteria and found the prevalence of major depressive disorder in two primary care settings was 5.4% (95% CI 2.1–8.7).

It is well known that physical illness and depression are major risk factors for suicidal ideation [25]. This study also supported this notion. Suicidal ideation associated with poor subjective health may have had a partial correlation with somatic symptoms from an accompanying depressive disorder in this study. One other study found that 45–95% of patients with depression worldwide report only somatic syndrome [26]. Therefore, some primary care patients might often visit local clinics to seek help for somatic symptoms of unknown origin without recognizing mental illness like a depressive disorder.

Some physicians in primary care settings did not realize that their patients suffered from depression; this non-recognition of suicidal ideations by primary care physicians is far more serious than depression. Moreover, even when physicians at local clinics correctly identified depression or suicidal ideations, most of the patients with suicidal ideation and a few patients with depression were only advised to see a psychiatrist but no arrangements for referral to a psychiatric service. Most primary care visitors in such a case would not see a psychiatrist on their own due to the social stigma to associated with having a mental illness. This trend implied that suicidal ideation and depression may be under-diagnosed and under-treated in primary care settings on a large scale. This implies that many primary care physicians are not educated enough to screen and manage patients at suicidal risk. According to a Korean study [27], the participating rate of primary care physicians in a suicide prevention education program was only 18.8%. The reason for such a low participation is because there is no suicide education program and a clinical practice guideline for suicide prevention in primary care supported by central government. On the contrary, some industrialized countries such Australia [11] and United Kingdom [12] have a clinical practice guideline and education program for suicide prevention in primary care.

The OECD also recommended that an expansion of the role of primary care in suicide prevention will be required to lower the high suicide rates in Korea [9]. The first step in addressing this issue should be education on suicide prevention in clinical practice, even though Korean primary care providers are very busy with patients who have acute and chronic physical illnesses. Given that 90% of local clinics in Korea are privately operated under fee-for-service payment of KNHI, screening and management of patient at suicidal risk by them can be done only if it gets financial and institutional support from the government.

This study has several limitations, which must be pointed out. Our sample is not representative of all visitors at local clinics in Korea because of the small sample size and convenience sampling of primary facilities involved in this study. However, we made our sample more representative by selecting two areas that included both urban and rural regions and by carrying out consecutive sampling of their primary care visitors. Moreover, we failed to classify the characteristics of non-respondents. Considering that the reasons for choosing not to take part in our survey included concerns about the release of personal information to the public and hostility towards any investigation of depression and suicidal ideations, some patients who suffered from these conditions might have been missed. Finally, physicians in this study were asked their recognition of suicidal idea or depression among their patients as a general question. It is necessary for development of suicide prevention policy in primary care that physicians will be asked it in reference to the individual patients from their clinic Nevertheless, this study is the first attempt to identify the prevalence of suicidal ideation and depression in primary care patients by physicians in Korean primary care settings.

## Conclusions

This study showed the prevalence of suicidal ideations and depression in primary care in South Korea. The rates identified in primary care settings are far higher than those in general population which was in agreement with the existing literature from other countries. However, many cases of suicidal ideation and depression might be under-diagnosed and under-treated in primary care. Therefore, physicians in primary care settings should be given education on recognition and management of depression and suicidal ideation in clinical practice.

## Abbreviations

OECD: Organization for Economic Co-operation and Development
PHC: Public Health Center
PHQ-9: Patient Health Questionnaire-9
KNHNES: Korean National Health & Nutrition Examination Survey
